# Assessing the neurological impact of vitamin B12 deficiency among the population of Riyadh, Saudi Arabia

**DOI:** 10.3389/fnut.2025.1635075

**Published:** 2025-08-19

**Authors:** Khalid A. Bin Abdulrahman, Abdulelah F. Alshehri, Faisal Marzouq Almutairi, Faisal Ali Almahyawi, Rayan Saleh Alzahrani, Osama Thamer Al-Ahmari, Khalid Alshammari, Turki Ahmed Aljuhani, Yazeed Yahya Alkadi, Mohammed Atiah Alisi

**Affiliations:** ^1^Department of Medical Education, College of Medicine, Imam Mohammad Ibn Saud Islamic University (IMSIU), Riyadh, Saudi Arabia; ^2^Department of Family Medicine, College of Medicine, Imam Mohammad Ibn Saud Islamic University (IMSIU), Riyadh, Saudi Arabia; ^3^Department of Family Medicine, Family Medicine Academy, Eastern Health Cluster, Khobar, Saudi Arabia; ^4^Faculty of Medicine, King Abdulaziz University, Jeddah, Saudi Arabia

**Keywords:** vitamin B12, cobalamin, nervous system, public awareness, Saudi Arabia

## Abstract

**Background:**

Vitamin B12 (cobalamin) deficiency can cause anemia, peripheral neuropathy, visual impairment and irreversible neurological damage. Little is known about public awareness of these neurological effects in Saudi Arabia. This study assessed awareness of the neurological impact of vitamin B12 deficiency among adults in Riyadh, Saudi Arabia.

**Methods:**

We conducted a population-based cross-sectional survey in Riyadh between March and April 2024. Adults aged ≥18 years were recruited via social media. Individuals affiliated with the health sector were excluded to reduce bias. A validated questionnaire assessed sociodemographics, awareness of vitamin B12, dietary sources, deficiency symptoms and prevention. Descriptive statistics and chi-square tests were used (*P* < 0.05).

**Results:**

Of 1,337 participants, 23 were excluded, leaving 1,314 respondents; 62.3% were female. Most (65.6%) recognized the importance of vitamin B12, 38.7% identified a dietary source, and 29.9% used supplements. Awareness of the neurological consequences of deficiency was reported by 63.4%. Women showed greater knowledge than men (*P* < 0.001), and employed participants or those in “other” occupations were more aware than students.

**Conclusion:**

Overall awareness of vitamin B12 and its neurological consequences was moderate. However, knowledge of dietary sources and prevention strategies was limited, and misperceptions (e.g., fruits and vegetables as sources) were common. Targeted public-health education and nutritional counseling are needed to address these gaps.

## Graphical Abstract

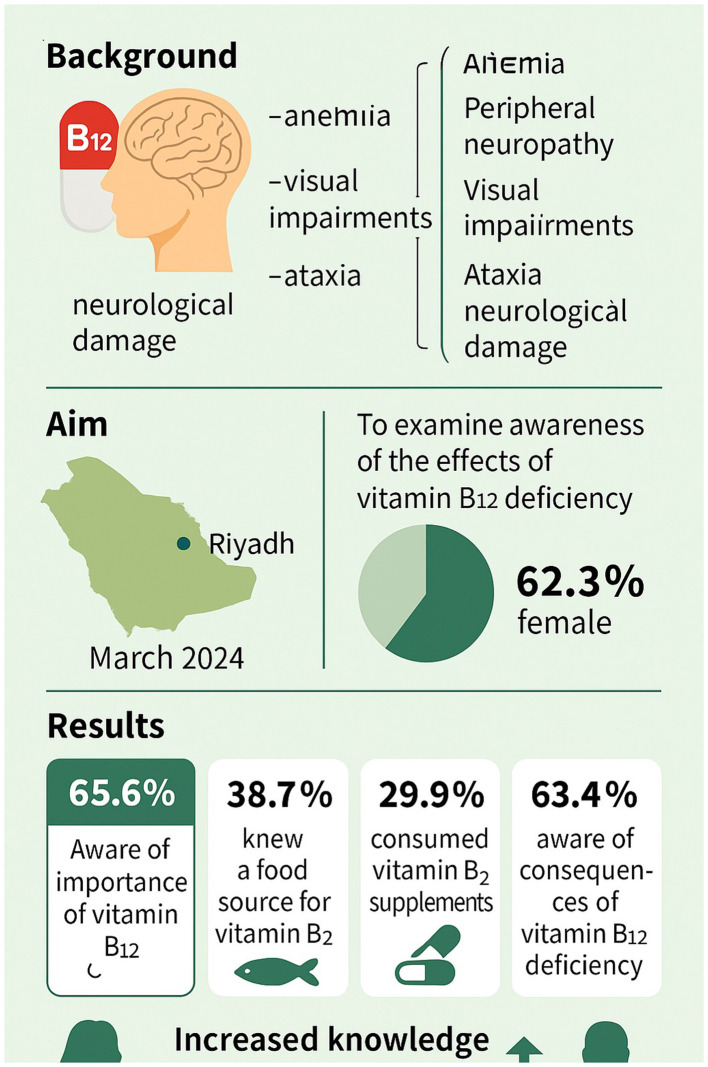

## 1 Introduction

Cobalamin, commonly known as vitamin B12, is a water-soluble vitamin naturally bound to proteins in food. It is absorbed in the distal ileum through a complex with intrinsic factor (IF), a glycoprotein secreted by gastric parietal cells. Once absorbed, vitamin B12 enters the bloodstream and is ultimately excreted in the feces. The biologically active forms involved in human metabolism are methylcobalamin and 5-deoxyadenosylcobalamin (1–3).

Vitamin B12 plays a vital role in DNA synthesis, red blood cell production, and the maintenance of neurological function, including myelination and neurotransmitter synthesis (4). It facilitates the conversion of homocysteine to methionine, a precursor to S-adenosylmethionine, a key methyl group donor for various biochemical processes. Gastric acid and proteases liberate B12 from food proteins, a step necessary for absorption.

Deficiency in vitamin B12 is a global public health concern and can result in irreversible neurological damage if not promptly identified and treated (4–6). Symptoms may include paresthesia, cognitive decline, mood disturbances, visual disturbances, and autonomic dysfunction (7). Risk factors include malnutrition, malabsorption syndromes, gastric atrophy, and dietary restrictions (e.g., vegetarianism).

Recommended dietary allowances (RDAs) vary by age and physiological state: 0.9 mcg/day for toddlers, 2.4 mcg/day for adults, 2.6 mcg/day during pregnancy, and 2.8 mcg/day for lactating women (5, 6). Individuals over 50 years or on restricted diets may require fortified foods or supplementation (6–10).

Treatment of deficiency often involves intramuscular injections of crystalline B12: 1 mg weekly for 8 weeks, followed by monthly maintenance for life (9).

Vitamin B12 deficiency significantly affects neurological health and quality of life but remains underrecognized. In many populations, including those in urban centers such as Riyadh, awareness of B12 deficiency remains low despite its high prevalence among at-risk groups such as the elderly, individuals with gastrointestinal disorders, and those adhering to restrictive diets. Public health initiatives targeting early detection and education are therefore critical to prevent long-term neurological complications and reduce healthcare burdens. Identifying knowledge gaps and symptoms may inform national health strategies and screening guidelines.

This study aimed to assess the neurological effects of vitamin B12 deficiency and the level of public awareness in Riyadh, Saudi Arabia.

## 2 Methods

### 2.1 Study design and settings

**Figure 1:**
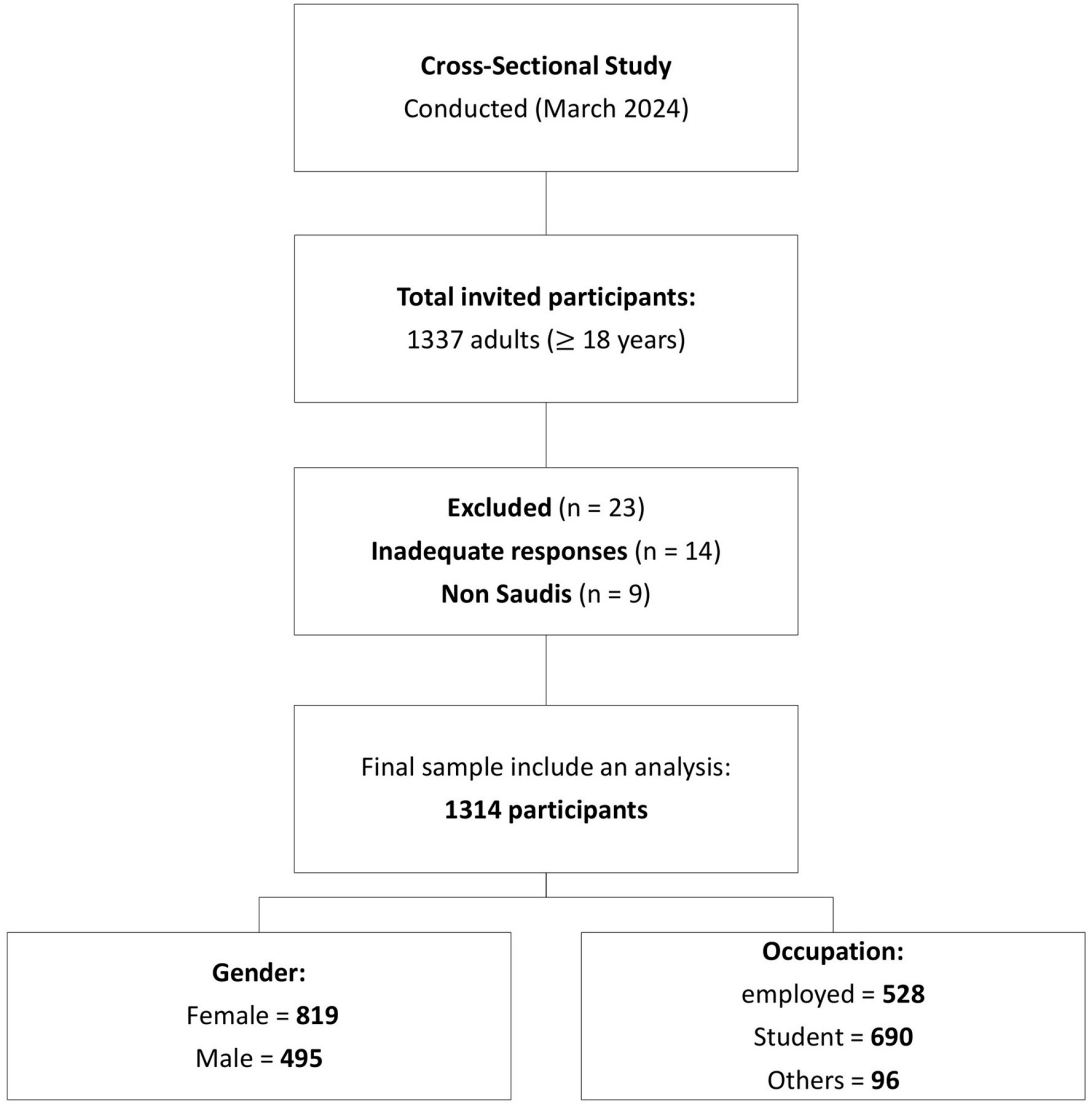
Flowchart illustrates the study design and population distribution.

A cross-sectional study was conducted in Riyadh, Saudi Arabia, from March 2024 to April 2024 to assess residents' awareness of the impact of vitamin B12 deficiency on the nervous system. Any residents aged 18 years and above were invited to participate in the survey. We excluded individuals associated with the health industry, including physicians, pharmacists, and others possessing prior knowledge of vitamin B12, using a question included in the questionnaire asking participants, “Are you affiliated with the health sector?” Those who answered “yes” were excluded to maintain study objectivity. Figure 1 illustrates the study design and population distribution. All procedures were performed according to the ethical principles of the Declaration of Helsinki. The survey link included a brief overview and detailed explanation. The participants were told that completing the survey constituted consent. All participant consent and data were collected with complete confidence throughout the study.

### 2.2 Sample size

Our sample size was determined using the Raosoft sample size calculator (Raosoft Inc., Seattle, Washington, USA). For a confidence level of 95% and a margin of error of 5%, a sample size of 384 participants was identified as the projected minimum advisable sample size for our survey.

### 2.3 Data collection

We used a validated assessment tool from a previously published study (11), which reported face and content validity conducted by domain experts and piloted the tool among 30 participants prior to full deployment. The tool was slightly modified to ensure cultural relevance and linguistic clarity for the Saudi population. Cronbach's alpha for the knowledge-related questions in our pilot was 0.81, suggesting good internal consistency.

The participants were approached via social media platforms with a link to the study questionnaire provided in Arabic and English. The authors included a consent form for the use of participant data in the research. Each participant provided informed consent according to the requirements of the university ethics committee for cross-sectional studies. The questionnaire included two sections: sociodemographic data and awareness of B12 deficiency effects on the nervous system. For analysis purposes, Participants answering “Yes” to three or more of the six knowledge items were classified as having “adequate awareness”, corresponding to a 50% correct response threshold—commonly used in public health awareness studies (12).

### 2.4 Data analysis

Frequency tables were computed to demonstrate demographic variables, and chi-square (χ^2^) tests were performed to compare awareness among groups. All the data were stored and sorted using Microsoft Excel version 2023, and the analysis was performed using the Statistical Package of Social Science (SPSS) version 29. A *P* < 0.05 was considered to indicate statistical significance. The IBM Statistical Package for the Social Sciences (SPSS), Windows version 26.0, was used to analyze the data, and Microsoft Excel 2016 was used to present the data in tables and graphs.

## 3 Results

A total of 1,337 adults aged 18 years and above were invited to participate. Of these, 23 individuals were excluded–14 due to inadequate responses and 9 for being non-Saudis—resulting in a final sample size of 1,314 participants included in the analysis.

Table 1 shows the relationships between demographic characteristics and self-reported knowledge about the effects of vitamin B12 deficiency on the nervous system. Compared with male participants, 198 of whom reported awareness of these effects, females displayed greater understanding, with 423 participants reporting awareness; this difference was statistically significant (*P* < 0.001). Awareness did not significantly differ depending on pregnancy status, with knowledge levels similar between women who were pregnant and women who were not pregnant (*P* = 0.177).

**Table 1:** Relationships between demographic characteristics and self-reported knowledge about the effects of vitamin B12 deficiency on the nervous system.

**Demographic characteristics**		**Knowledge**		***P* value**
		**Yes**	**No**	
Sex	Male	198	306	< 0.001
	Female	423	410	
If you are female, are you pregnant?	Yes	10	4	0.177
	No	413	406	
Nationality	Saudi	573	668	0.524
	Non-Saudi	48	48	
Occupation	Employed	170	162	<0.001
	Student	325	446	
	Other	126	108	

Association between demographic characteristics and self-reported knowledge of the neurological effects of vitamin B12 deficiency. This table presents the number of participants who reported having or lacking knowledge about the neurological consequences of vitamin B12 deficiency, stratified by sex, pregnancy status (among females), nationality, and occupation. Statistical significance was assessed using chi-square tests; P < 0.05 indicate significant associations.

Knowledge of the effects of vitamin B12 deficiency on the nervous system across nationalities was comparable, with Saudi and non-Saudi participants showing no significant difference (*P* = 0.524). However, occupation was an important factor; employed individuals and those in the “other” occupational category demonstrated greater awareness than students did (*P* < 0.001).

As presented in Table 2, self-reported knowledge of vitamin B12 varied among the participants. A large proportion of the participants had heard about vitamin B12, with 1,194 indicating “Yes” (89.3%), with 143 participants (10.7%) reporting that they had not heard about it. A total of 847 participants were aware of vitamin B12 deficiency (63.4%), whereas 490 participants were not aware of vitamin B12 deficiency (36.6%). Fewer than half of the participants (621 [46.4%]) knew about the effects of vitamin B12 deficiency on the nervous system, whereas 716 (53.6%) participants were not aware of these effects.

**Table 2:** Self-reported knowledge of vitamin B12 among the participants.

**Item**		***N***	***N*%**
Have you ever heard about vitamin B12?	Yes	1,194	89.3%
	No	143	10.7%
Do you know the effects of vitamin B12 deficiency?	Yes	847	63.4%
	No	490	36.6%
Do you know the effects of vitamin B12 deficiency on the nervous system?	Yes	621	46.4%
	No	716	53.6%
Do you know the importance of vitamin B12?	Yes	877	65.6%
	No	460	34.4%
Are you a strict vegetarian or vegan?	Yes	83	6.2%
	No	1,254	93.8%
Do you know any food source for vitamin B12?	Yes	518	38.7%
	No	819	61.3%
Do you know how to prevent vitamin B12 deficiency?	Yes	357	26.7%
	No	980	73.3%
Do you take any vitamin B12 supplements?	Yes	400	29.9%
	No	937	70.1%

Participants' self-reported knowledge and practices related to vitamin B12. This table summarizes the responses of study participants to questions regarding awareness, knowledge, dietary habits, and supplementation related to vitamin B12. Frequencies and percentages are provided for each response category.

Awareness of the importance of vitamin B12 was slightly higher, with 877 (65.6%) participants who recognized the importance of this vitamin and 460 (34.4%) who were unaware. Only a small fraction of the participants were strict vegetarians or vegans (83 participants [6.2%]), as opposed to the majority of participants (1,254 participants [93.8%]) who were not vegetarian or vegan. Only 38.7% knew a food source of vitamin B12; 61.3% did not.

Only 357 (26.7%) participants were informed about methods for preventing vitamin B12 deficiency, and a substantial majority of 980 (73.3%) participants did not know of any prevention methods. Only 29.9% took B12 supplements, while 70.1% did not.

**Figure 2:**
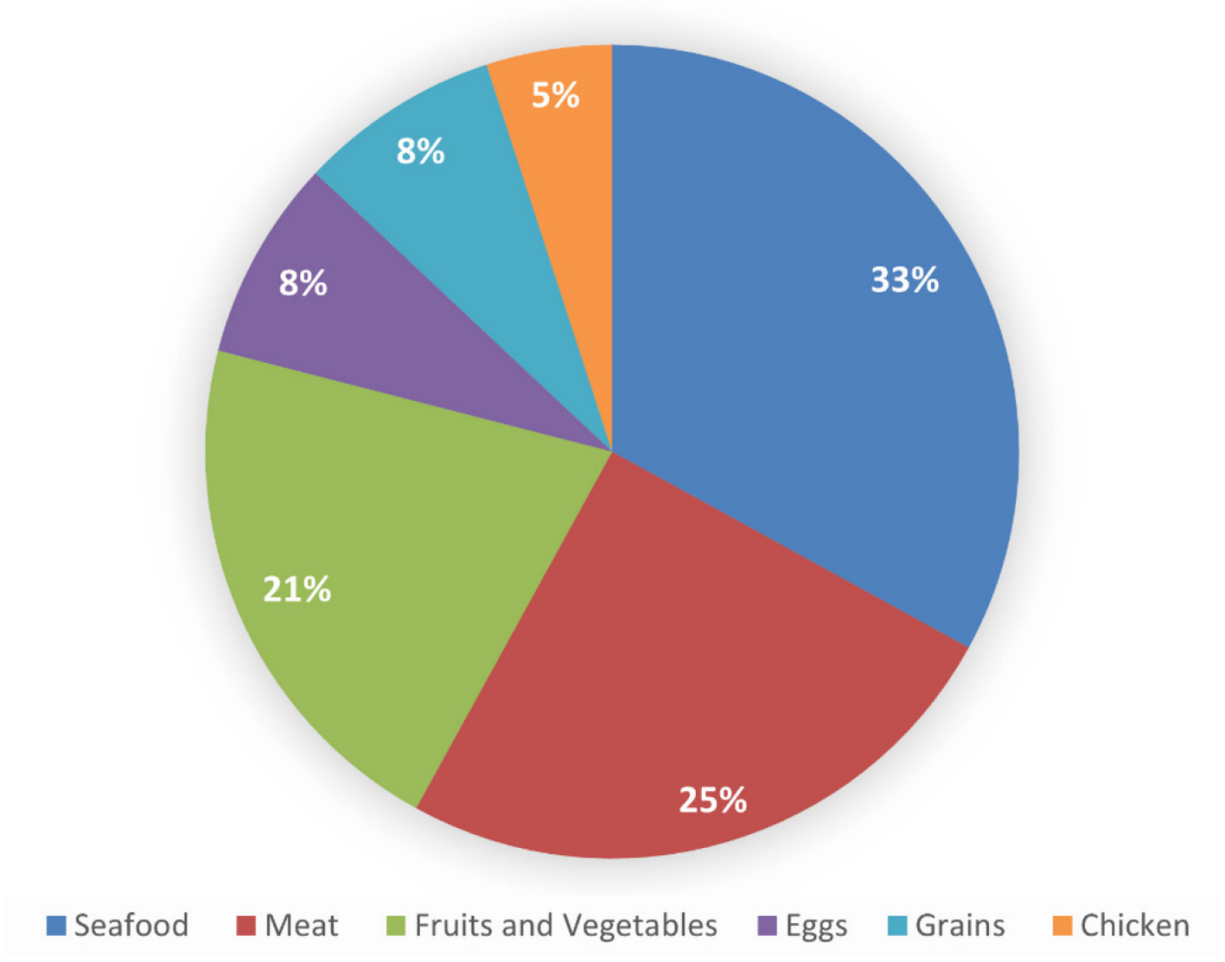
Food sources rich in vitamin B12.

As shown in Figure 2, when the participants were asked to choose a food source that they thought was rich in vitamin B12, 438 (32.8%) participants identified seafood as being rich in vitamin B12. Meat was also commonly recognized as a source of vitamin B12 (336 participants [25.1%]). A substantial number of participants incorrectly identified fruits and vegetables as rich in vitamin B12, with 274 (20.5%) participants choosing this option. Grains and eggs were identified by similar proportions of the study population, with 109 (8.2%) choosing grains and 108 (8.1%) choosing eggs. Finally, chicken was the least selected option, with 72 participants (5.4%) considering it a food source rich in vitamin B12.

As shown in Table 3, just over half of the study participants, 712 individuals (53.3%), reported having been tested for vitamin B12 levels, whereas a slightly smaller group of 625 individuals (46.7%) had not tested. Among participants who were tested, 364 participants (51.1%), which was a slight majority, had results indicating a below-normal vitamin B12 level. A comparable number of participants had normal vitamin B12 levels, with 333 individuals (46.8%), and a small minority had levels above normal, with 15 participants (2.1%).

**Table 3:** Previous measurements of vitamin B12 in the study participants.

**Variable**		**N**	**N%**
Have you ever been tested for vitamin B12 levels?	Yes	712	53.3%
	No	625	46.7%
If yes, what were the results?	Normal	333	46.8%
	Below normal	364	51.1%
	Above normal	15	2.1%

History of vitamin B12 level testing among participants. This table shows the proportion of participants who had previously undergone vitamin B12 testing and their reported test results (normal, below normal, or above normal). Results are presented as frequencies and percentages.

**Figure 3:**
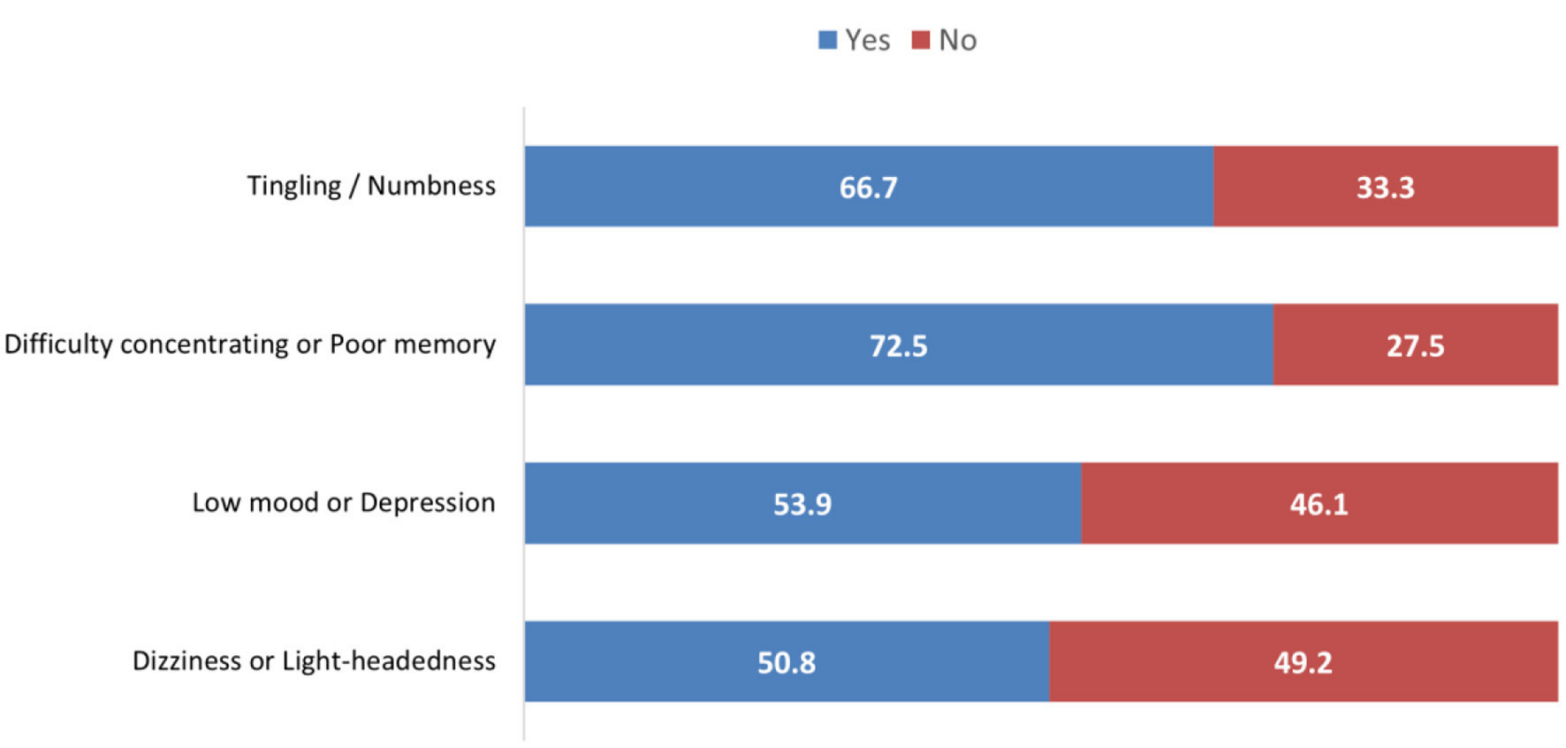
Awareness of vitamin B12 deficiency symptoms.

As depicted in Figure 3, awareness of vitamin B12 deficiency symptoms among the participants varied. A total of 892 (66.7%) participants were aware of “tingling or numbness” as a symptom. The symptom “difficulty concentrating or a poor memory” had the highest level of awareness, with 969 participants (72.5%) recognizing this symptom. Over half of the participants (720 [53.9%]) were aware of “low mood or depression” as a symptom of vitamin B12 deficiency. Awareness of “dizziness or lightheadedness” as a symptom was split among participants, with 679 (50.8%) answering “Yes” and 658 (49.2%) answering “No”.

## 4 Discussion

Vitamin B12 is essential for erythropoiesis, cellular metabolism, and neurological integrity. Early identification and intervention are critical to prevent irreversible nerve damage. Despite the importance of this vitamin, studies on public awareness, particularly in the Middle East, remain limited. This study addresses that gap by evaluating knowledge levels and associated neurological symptoms in Riyadh, Saudi Arabia.

Most participants (62.3%) were females. The convenience of the survey may explain why female respondents were predominant. Most participants (89.3%) were aware of vitamin B12. This finding was similar to that of the study conducted by AlMufarej et al. (13), which reported a rate of 77.9%, and that of the study conducted by Alqurashi et al. (12), which reported a rate of 87.7%. The respondents reported a relatively high level of awareness of the repercussions of vitamin B12 deficiency, with 64% of individuals reporting awareness (12). Our findings were comparable, with 63.4% of respondents reporting awareness of these consequences. A separate study conducted in Saudi Arabia revealed that 78% of participants demonstrated awareness of the importance of vitamin B12 and the potential repercussions of vitamin B12 deficiency (13).

The majority of participants (32.8%) in this study identified shellfish as a food high in B12, whereas meat was selected by 25.1% of participants. In the study by Alqurashi et al. (12), seafood was the category most frequently chosen by the participants (55%), followed by animal items (22%). In contrast, another prior investigation reported that 41% of participants were aware of food sources that are rich in vitamin B12 (14). In this study, a notable misconception was identified, as 20.5% of participants incorrectly reported fruits and vegetables as dietary sources of vitamin B12. This finding underscores a critical gap in nutritional knowledge, given that vitamin B12 is naturally found only in animal-derived foods such as meat, fish, eggs, and dairy products. Mistaking plant-based foods as B12 sources reflects a broader nutritional misconception, posing risks for vegetarians and vegans. This highlights the need for targeted public health education campaigns to enhance awareness about essential nutrient sources and promote accurate dietary practices. A French study yielded a high and consistent consumption rate of seafood—including fatty fish—among individuals eating seafood at least twice weekly (15).

Slightly more than half (53.3%) of the participants in this study reported undergoing testing for vitamin B12 levels. Among the individuals who underwent testing, a small majority (51.1%) had below-average levels of vitamin B12, 46.8% fell within the normal range, and 2.1% had above-average levels. AlMufarej et al. (13) reported that approximately 55.6% of subjects had vitamin B12 levels within the normal range.

A majority of participants (89.3%) reported awareness of vitamin B12, consistent with findings from other local studies (12, 13). Approximately 63.4% recognized the neurological consequences of deficiency, including cognitive difficulties, paresthesia, mood disturbances, and dizziness—Symptoms such as paresthesia, mood disturbances, dizziness, mental illness, tremors, insomnia, impaired concentration and other nervous-system problems are often misattributed to other conditions (16). In the study by Shah et al. (17) on blood donors in a rural tertiary hospital in India, 12% of participants reported some indicators of vitamin B12 deficiency, in contrast to prior results. Our findings indicate that females (*P* < 0.001) and employed individuals (*P* < 0.001) presented considerably greater levels of comprehension and awareness regarding vitamin B12 deficiency than males and students did. Furthermore, an investigation carried out in Jeddah corroborates this finding (18). In addition, Khan et al. (19) reported that almost 90% of female college students in Oman were aware that sunlight is the main source of vitamin D. AlGarni et al. (20) reported that female awareness scores were significantly greater (*P* = 0.020).

Based on the observed gaps in awareness and understanding of vitamin B12 deficiency, particularly among specific demographic groups such as youth and vegetarians, we recommend the implementation of targeted public health campaigns and educational initiatives. These strategies should focus on increasing awareness about the neurological consequences of vitamin B12 deficiency, promoting dietary sources rich in cobalamin, and encouraging routine screening in at-risk populations. Community-based awareness programs, collaboration with primary care providers, and integration of nutrition education into school and university curricula could significantly enhance public knowledge and preventive behavior.

Health authorities should use mass and social media to share culturally appropriate, evidence-based content about B12 deficiency and neurological risks. These efforts can include public service announcements, influencer-led campaigns, short educational videos, and infographics that highlight early warning signs of deficiency, neurological risks, and the importance of regular testing. Particular attention should be paid to correcting common misconceptions, such as the belief that fruits and vegetables are adequate sources of vitamin B12. Additionally, targeted messaging should reach vulnerable groups, such as elderly individuals, strict vegetarians, and individuals with gastrointestinal conditions that impair absorption.

Maintaining adequate vitamin B12 levels requires both awareness and access to proper dietary sources and supplements. To prevent deficiency, the public should be encouraged to include B12-rich foods such as meat, dairy, eggs, and fortified cereals in their daily diet. For those at risk of low intake—such as vegans or individuals with malabsorption syndromes—healthcare providers should advocate for regular screening and supplementation with oral or intramuscular vitamin B12 as needed. Pharmacies and primary care centers can also play a critical role by offering counseling services and distributing educational brochures during routine visits. By integrating these interventions into public health policy and clinical practice, the long-term burden of neurological complications from B12 deficiency can be effectively mitigated.

To evaluate the representativeness of our study sample, we compared its demographic profile with available statistics from the General Authority for Statistics in Saudi Arabia. According to the 2022 Riyadh Region Demographic Survey, the gender distribution in the general adult population is ~55% male and 45% female, whereas our study sample comprised 62.3% females and 37.7% males. This overrepresentation of female participants may be attributed to the online, voluntary nature of survey recruitment, which often sees greater engagement from women in health-related topics. Additionally, in terms of occupational status, our sample included a substantial proportion of students (52.5%) and employed individuals (40.2%), which may not fully align with the workforce composition of Riyadh, where employment rates are generally higher among adults aged 25–54. Nationality-wise, the overwhelming majority of participants were Saudi (94.6%), which is consistent with local sampling priorities, though the Riyadh region includes a notable proportion of non-Saudi residents (~40% overall). These discrepancies highlight a potential sampling bias and underscore the need for caution when generalizing the results to the broader Riyadh population.

## 5 Limitations

The study relied on social media platforms for participant recruitment, which may have introduced selection bias, potentially leading to an overrepresentation of tech-savvy, educated, or female individuals. This recruitment method may limit the generalizability of the findings to the broader population. Additionally, the use of self-reported data may be prone to misclassification or underreporting biases, potentially affecting the accuracy of reported knowledge and behaviors.

## 6 Conclusion

While a moderate level of knowledge was observed, significant gaps remain—particularly in identifying dietary sources and symptoms of deficiency. Female participants and employed individuals demonstrated higher awareness than other groups. These findings underscore the need for targeted educational interventions and public health initiatives to improve awareness, prevention, and early detection of vitamin B12 deficiency.

## Data Availability

The raw data supporting the conclusions of this article will be made available by the authors, without undue reservation.
